# Supplementary figures and images for: Identifying Keystone Species in the Human Gut Microbiome from Metagenomic Timeseries Using Sparse Linear Regression
Source: PLoS One. 2014 Jul 23;9(7):e102451. doi: 10.1371/journal.pone.0102451 (PMC4108331; doi:10.1371/journal.pone.0102451)

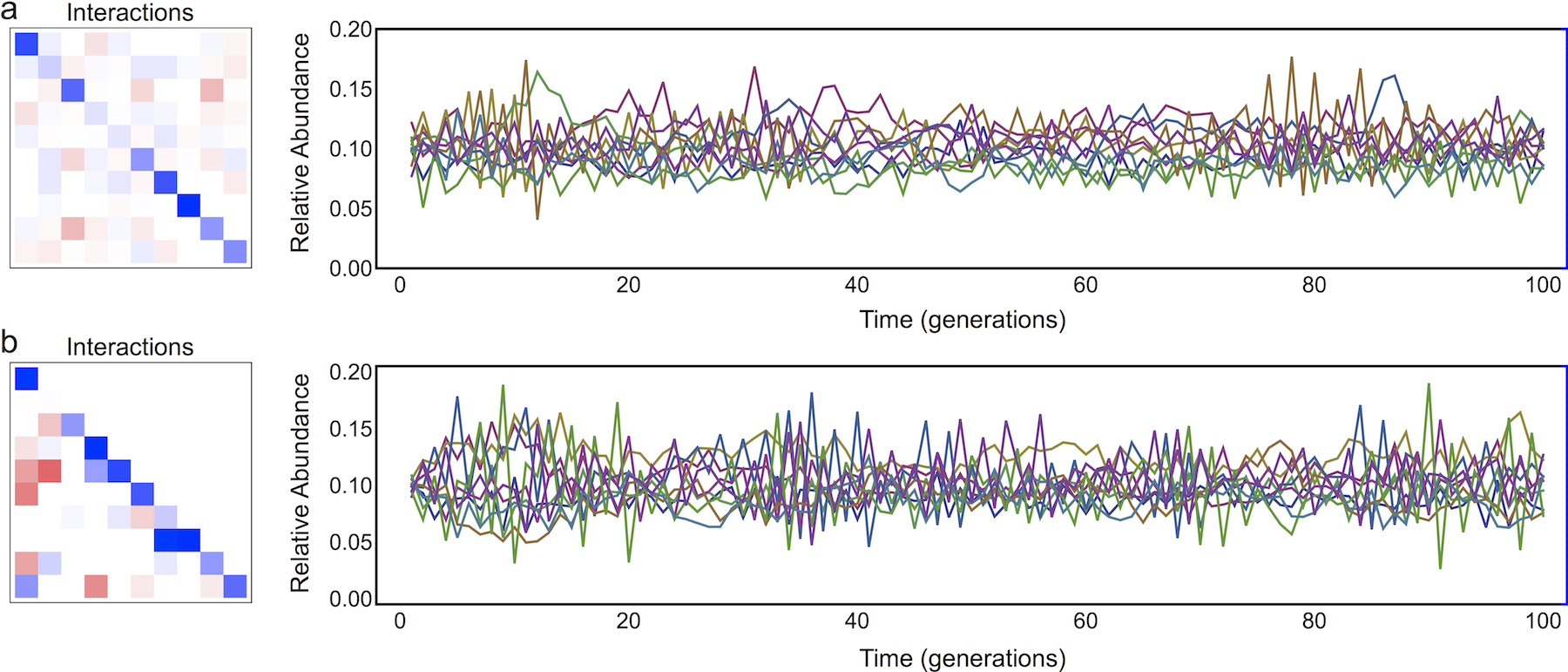

Supplement: Figure S1 — Example Lotka-Volterra Time Series. Representative simulations of Lotka-Volterra time series with a) a symmetric interaction matrix and b) an asymmetric interaction matrix. (TIFF) [file pone.0102451.s002.tiff]

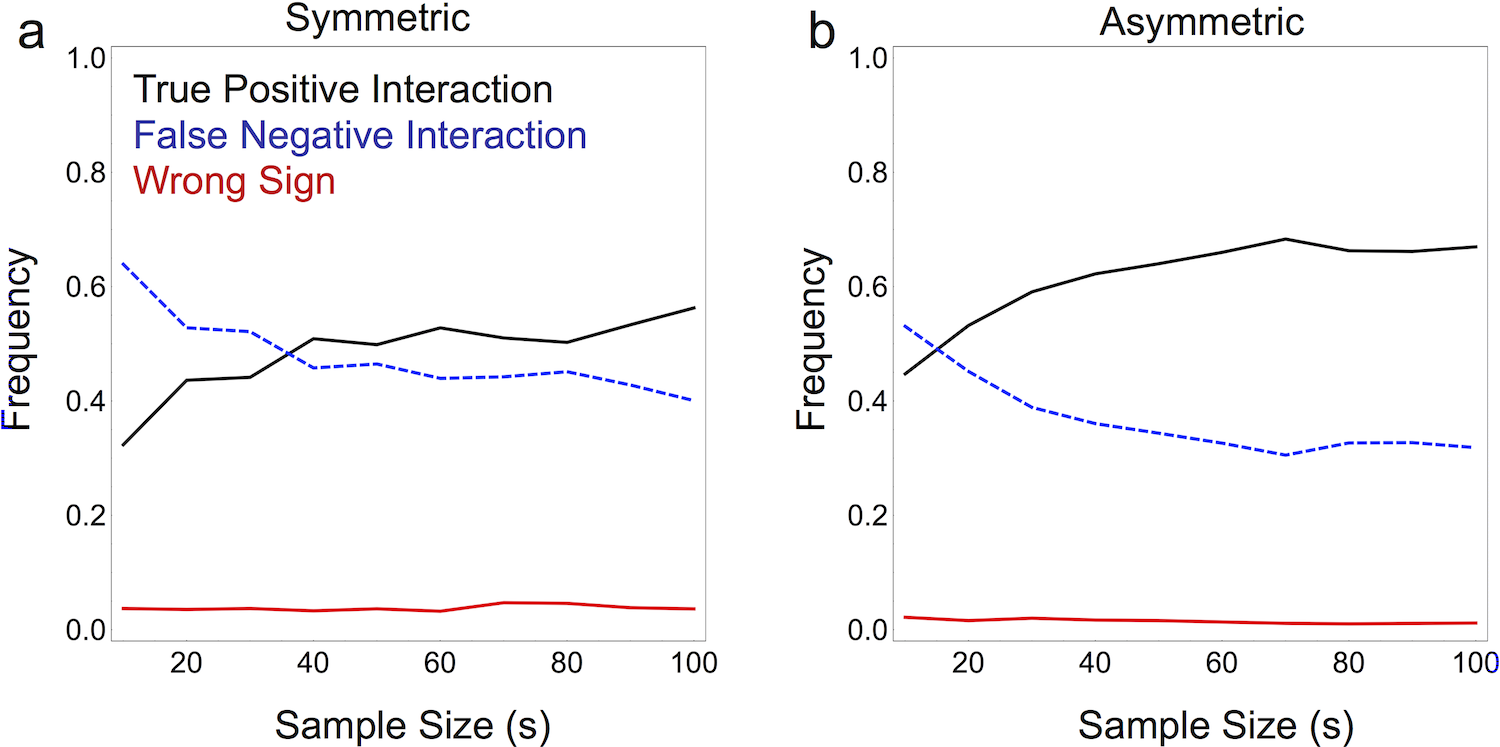

Supplement: Figure S2 — Errors in Interaction Topology as a Function of Sample Size. The errors in inferred interaction topologies as a function of sample size for a) a symmetric interaction matrices and b) an asymmetric interaction matrices. The black line represents the true positive rate, the dashed blue line represents the false negative rate, and the red line represents the rate of sign errors. (TIFF) [file pone.0102451.s003.tiff]

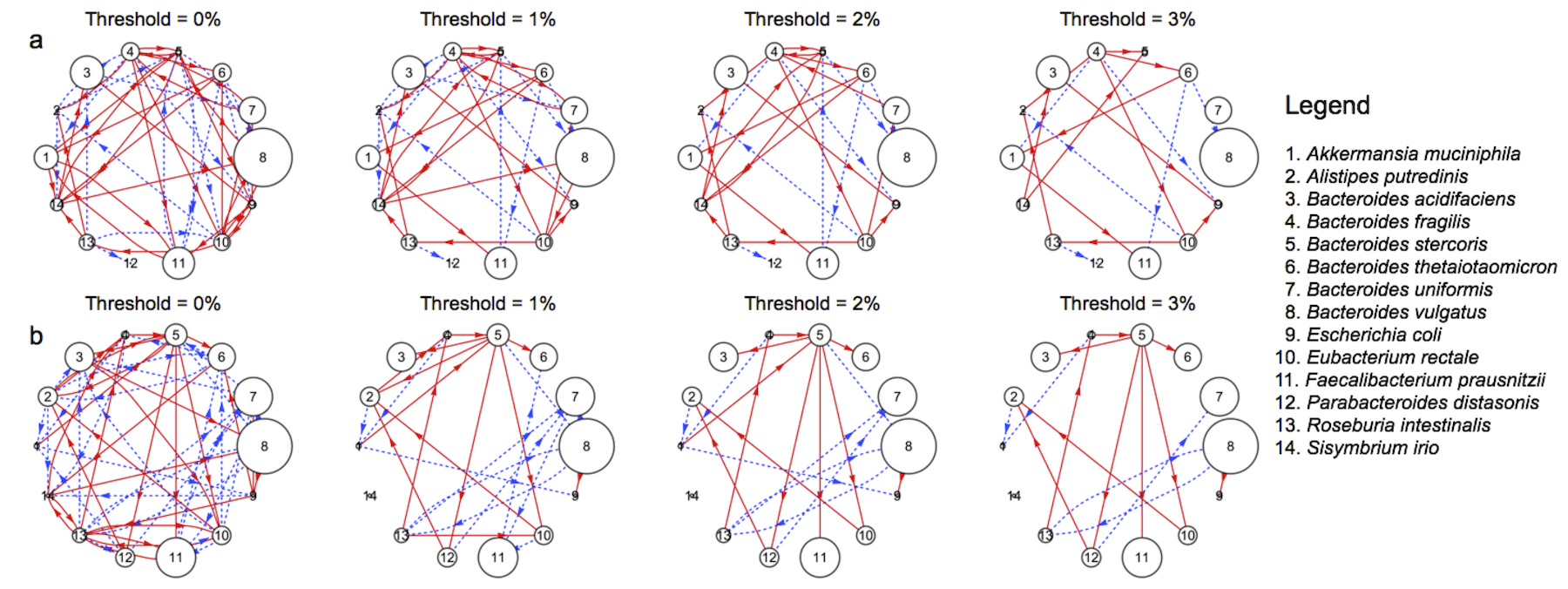

Supplement: Figure S3 — Interaction topologies of abundant species in the guts of two individuals using different prediction error thresholds. The size of a node denotes the median relative species abundance, beneficial interactions are shown as solid red arrows, and competetive interactions are shown as dashed blue arrows. The 14 species included in the model were obtained by taking the union of the top 10 most abundant species from individuals a and b. (TIFF) [file pone.0102451.s004.tiff]
